# Physiological determinants of heterosis in hybrid rice (*Oryza sativa* L.): identifying the optimal phenotyping stage for yield prediction

**DOI:** 10.3389/fpls.2026.1871769

**Published:** 2026-07-29

**Authors:** Faraz Azeem, Jauhar Ali, Tonette P. Laude, Sta Pompe. C. Cruz, Eureka M. Ocampo, Seyed Mahdi Hosseinian Khatibi, Jose E. Hernandez

**Affiliations:** 1 Rice Breeding Innovation Department, International Rice Research Institute, Los Baños, Philippines; 2 Institute of Crop Science, College of Agriculture and Food Science, University of the Philippines Los Baños, Laguna, Philippines

**Keywords:** BLUP, CMS, heterosis, hybrid rice, multi-environment trials, physiological phenotyping, source-sink coordination, TGMS

## Abstract

**Introduction:**

Hybrid rice productivity depends on heterosis, yet the developmental stage at which physiological traits become sufficiently integrated to reliably predict grain yield across diverse environments remains poorly understood. Although physiological phenotyping is increasingly used in crop improvement, the biological basis for selecting the most informative developmental stage for early yield prediction has received limited attention.

**Methods:**

A panel of 39 rice genotypes, including hybrids, parentals, and check varieties, was evaluated across four environments. Fourteen physiological and agronomic traits were measured at the early (30 days after sowing, DAS) and mid-vegetative (60 DAS) stages. Best linear unbiased predictors (BLUPs) derived from mixed models were used to estimate genotype performance from an unbalanced multi-environment dataset, followed by multivariate analysis, leave-one-season-out cross-environment validation, and development of a physiology-based selection index.

**Results:**

BLUP-based multivariate analyses revealed a distinct productivity axis separating hybrids from parental lines, indicating that heterosis was associated with coordinated physiological and structural development rather than enhancement of individual traits alone. High-performing two-line and three-line hybrids exhibited broadly similar physiological patterns, suggesting shared physiological mechanisms underlying superior yield performance. Traits measured at 60 DAS consistently outperformed those measured at 30 DAS for predicting grain yield across environments. Chlorophyll content (SPAD60) and plant height (PH60) emerged as the most robust predictors, while stomatal conductance (FLGS60) provided complementary physiological information. Leave-one-season-out validation demonstrated that mid-vegetative traits achieved the highest predictive performance (r ≈ 0.38), whereas early-stage traits showed weak and inconsistent predictive ability. Combining early and mid-stage measurements did not improve prediction. A selection index based on key 60 DAS traits achieved a 39.08% yield advantage over the population mean.

**Conclusion:**

This study demonstrates that the mid-vegetative stage represents a critical developmental transition during which physiological stabilization, canopy development, biomass consolidation, and coordinated source-sink function converge to support reliable yield prediction. Rather than identifying individual predictor traits alone, our findings establish a developmentally informed physiological framework explaining why 60 DAS provides the most biologically informative stage for hybrid evaluation across environments. This framework offers a practical basis for physiology-guided phenotyping, cost-efficient early hybrid selection, and improved decision-making in hybrid rice breeding programs.

## 1 Introduction

Rice (*Oryza sativa* L.) is one of the most important staple crops globally, providing calories for more than half of the world’s population and playing a central role in food security across Asia and Africa (Birla et al., 2017; Shiferaw et al., 2013). In the face of rapid population growth, shrinking arable land, and climate variability, accelerating genetic gain in rice remains a major goal of crop improvement (Hussain et al., 2020; Sabar et al., 2024). Hybrid rice technology, which exploits heterosis, has consistently delivered significant yield advantage over inbred and hybrid checks (15-30% or more) and continues to be integral to breeding strategies aimed at maximizing productivity (Virmani, 1997; Yuan, 2017). Despite advances in hybrid breeding, the physiological mechanism and early phenotypic signal that reliably predict ultimate grain yield across environments remain insufficiently understood, especially under diverse field conditions. Hybrid rice seed is mainly produced using two breeding systems: the three-line cytoplasmic male sterility (CMS) system and the two-line thermosensitive genic male sterility (TGMS) system (Li et al., 2007; Siddiq and Ali, 1999). The three-line system involves male-sterile, maintainer, and restorer lines, while the two-line system uses environmentally sensitive TGMS lines whose sterility is regulated by temperature or photoperiod (Virmani, 2003; Fan and Zhang, 2018; Siddiq and Ali, 1999; Ali and Siddiq, 1999). Two-line system offers advantages in seed production flexibility and broader parental combination, making it increasingly attractive in global breeding programs (Azeem et al., 2025). However, the relationships among early phenotypic traits, physiological performance, and grain yield in these two systems remain poorly characterized under multi-season field conditions, limiting breeders’ ability to prioritize traits for early selection. Recent technological advances have significantly improved high-throughput phenotyping capabilities, enabling rapid, non-destructive measurements of physiological and canopy traits directly in the field. For example, handheld and proximal sensing devices such as SPAD chlorophyll meters, normalized difference vegetation index (NDVI) sensors, and porometers are now widely used to capture indicators of chlorophyll values, canopy vigor, and stomatal conductance, respectively. Studies have demonstrated that these tools can capture meaningful variation in physiological architecture and are associated with yield and biomass across diverse crop species (Araus and Cairns, 2014; Visakh et al., 2024). Specifically, NDVI and other vegetation indices have been repeatedly shown to correlate with yield in rice and other cereal crops, demonstrating their utility as indirect selection tools in breeding programs (Zhao et al., 2023; Phyu et al., 2020). Photosynthesis potential, leaf chlorophyll content, canopy structure, and stomatal conductance are among the physiological factors that influence biomass production and yield potential (Fan and Zhang, 2018; Ort et al., 2015). Despite these technological advances, a critical gap remains; most existing studies have focused on trait correlations measured at specific developmental stages or use remote sensing approaches under limited environmental conditions, such as canopy spectral indices derived from satellite imagery, with moderate success (Kurihara et al., 2023), but their applicability as early selection tools in hybrid breeding across multiple environments is not sufficiently validated. Moreover, while comprehensive reviews have highlighted machine learning and remote sensing methods for rice yield prediction and forecasting, they also emphasize persistent challenges such as model generalizability and limited ground-truth physiological data (Van Dijk et al., 2021; Angidi et al., 2025).

Early phenotyping offers significant advantages for breeding because it can reduce the cost and duration of selection cycles if reliable predictive traits are identified (Reynolds et al., 2019; Slater et al., 2017), during vegetative growth stages such as plant height, number of tillers, leaf number, and early canopy metrices reflect early vigor and biomass potential, which are often linked to resource acquisition and yield formation (Fields et al., 2014). Physiological traits measured at early stages, such as chlorophyll and stomatal conductance, reflect underlying plant function and stress responses that may influence reproductive success, especially in multi-environment field trial conditions and across different rice hybrid systems, and remain unexplored. Filling this gap is crucial for integrating phenomics into the practical breeding pipeline. The environmental inconsistency poses a major challenge in recognizing reliable phenotypic predictors of yield and its components. Genotype-by-environment (G×E) interactions can obscure trait-yield relationships, underscoring the need for multi-environment trials and robust statistical methods that partition genetic and environmental effects. Mixed models and best linear unbiased prediction (BLUP) have become standard tools for analyzing unbalanced multi-environment data and disentangling genetic signal from noise, thereby providing more accurate estimates of genotype performance and trait contributions to yield variation (Piepho et al., 2008; Xu et al., 2024). Such approaches are particularly significant in complex field datasets where trait measurements and genotype performance are influenced by seasonal and environmental fluctuations. Physiological traits associated with photosynthesis and canopy structure, such as chlorophyll content measured by SPAD meters, vegetation indices such as NDVI, and stomatal conductance, are promising candidates for early yield prediction. Chlorophyll content is widely recognized as a representation of leaf nitrogen and photosynthetic efficiency, while NDVI captures canopy architecture and light interception. Stomatal conductance is a key regulator of gas exchange and water use, with substantial implications for carbon assimilation and yield stability (Roche, 2015; Schulze et al., 1994). Integration of these physiological traits with agronomic traits such as plant height, tillering, and phenological timing may provide a more holistic understanding of yield determination and a pathway toward early selection. Most prior work has either focused on single environments or on end-of-season spectral data, limiting practical breeding insights. Furthermore, the potential differences in early trait expression between two-line and three-line hybrid systems remain largely unquantified. Understanding whether and how early physiological behavior differs between these hybrid types could inform hybrid profiling and targeted selection strategies, providing new insights for breeding programs. The objectives of this study were to: (i) quantify genetic variation and repeatability of early physiological and agronomic traits across environments; (ii) identify relationships between early phenotypic traits and final grain yield; (iii) evaluate the predictive ability of traits measured at different developmental stages across environments; and (iv) compare phenotypic patterns between two-line and three-line hybrid rice systems.

## 2 Materials and methods

### 2.1 Experimental sites and plant materials

Field experiments were conducted at the International Rice Research Institute (IRRI), Zeigler Experimental Station, Los Baños, Laguna, Philippines (14°13′N, 121°15′E; 21 m above sea level), and at Tublay, Benguet (16.52° N, 120.64° E; 1,072 m above sea level). Across four cropping seasons, IRRI wet season 2022 (IRRIWS_2022), IRRI dry season 2023 (IRRIDS_2023), IRRI dry season 2024 (IRRIDS_2024), and Tublay wet season 2022 (TWS_2022). Tublay experiences a low mean temperature (~24°C), thereby ensuring fertility expression in TGMS lines. A total of 39 unique genotypes were evaluated across four environments, resulting in 163 plot-level observations due to unbalanced testing. The panel consisted of two-line and three-line rice hybrid systems, including experimental test F_1_ hybrids, their parental lines, and commercial check varieties. The hybrid entries included two-line hybrids (IR144693H and IR142464H) and three-line F₁ hybrids (IR139526H, IR138766H, and IR145850H). The parental lines comprised thermosensitive genic male sterile (TGMS) lines, pollen parents, maintainer (B) lines, and restorer (R) lines. The commercial check varieties included inbred checks (IRRI247, IRRI154, and IRRI192) and hybrid checks (AZ33DT, NSICRC540H, NSICRC132H, SL-12H, and SL-19H). The field trials were conducted using a randomized complete block design (RCBD) with two replications for the cropping season IRRI wet season 2022 (IRRIWS_2022) and Tublay wet season 2022 (TWS_2022), and three replications for the IRRI dry season 2023 (IRRIDS_2023) and IRRI dry season 2024 (IRRIDS_2024), respectively. Each experimental plot measured 7.2 m^2^ and consists of 10 rows × 18 hills, with plants spaced at 20 × 20 cm. Due to breeding program logistic, the set of genotypes evaluated was not identical across seasons, resulting in an unbalanced multi-environment trial (MET). Specifically, 22, 10, 15, and 18 genotypes were evaluated in four environments, respectively, resulting in 163 plot-level observations. During the IRRI wet season 2022 (WS2022), the experiment was affected by a severe typhoon event that reduced grain yield; however, most physiological and agronomic traits had been recorded prior to the disturbance and were retained for analysis.

### 2.2 Crop management and field practices

**Figure 1:**
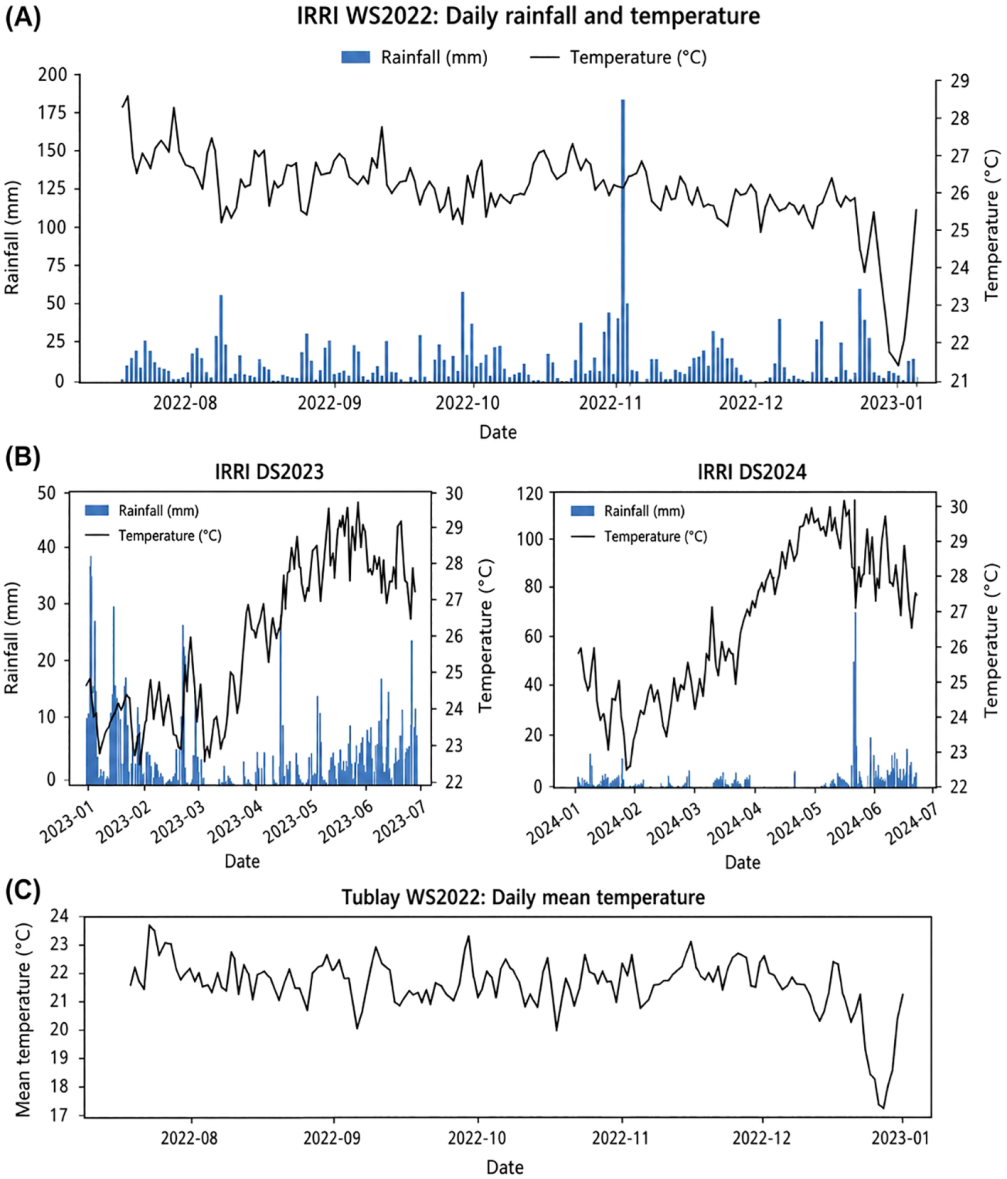
Seasonal variation in temperature and rainfall across experimental environments. Daily temperature (°C) and rainfall (mm) patterns are shown for IRRI wet season 2022 (WS2022) **(A)**, IRRI dry seasons 2023 (DS2023) and 2024 (DS2024) **(B)**, and Tublay wet season 2022 (WS2022) **(C)**. Rainfall is presented as bars and temperature as a line for IRRI environments, whereas only temperature is shown for Tublay due to data availability. The IRRI wet season exhibited high rainfall variability, including extreme events, whereas dry seasons showed comparatively stable temperature and lower rainfall fluctuation. Tublay environment was characterized by consistently lower temperatures. These patterns highlight the contrasting environmental conditions across sites and seasons. Climatic data were recorded on a daily basis. Temperature represents mean daily air temperature, and rainfall represents total daily precipitation.

Soil physicochemical properties were analyzed prior to sowing, and seasonal climatic data are provided in **Supplementary Table 1**. Daily climatic data, including temperature and rainfall, were recorded for each environment and are summarized in **Figure 1**. Seeds were subjected to heat treatment at 50 °C for 72 h in a hot air oven to break the seed dormancy. Seeds were then sown in seedling trays measuring 15 × 8 cm, and seedlings were transplanted into the field 21 days after sowing (DAS). Fertilizer was applied at the rate of 120-30-30–5 kg ha^-^¹ (N-P-K-Zn) across all seasons following standard IRRI agronomic recommendations. Nitrogen fertilizer was applied in split doses at transplanting, tillering, and panicle initiation stages, Standard crop management practices, including manual weeding, pest control, and removal of off-type plants, were implemented uniformly across all trials, respectively.

### 2.3 Phenotyping procedure

Phenotypic measurements were conducted in accordance with the Standard Evaluation System for Rice guidelines developed by IRRI (International Rice Research Institute (IRRI), 2014). For each plot level, five representative plants were randomly selected and tagged for repeated observation throughout the crop growth cycle.

### 2.4 Early vegetative traits

Early vegetative traits were measured at 30 days after sowing (30 DAS) and 60 days after sowing (60 DAS) to capture early canopy establishment and vegetative vigor. Plant height (PH30 and PH60; cm) was measured from the soil surface to the tip of the tallest leaf blade. Tiller number (NT30 and NT60) was counted as the number of active tillers per plant. Stem length (SL30, SL60; cm) measured from the soil surface to the base of the uppermost leaf sheath. Leaf number (LN30 and LN60) represented the total number of fully expanded leaves per plant.

### 2.5 Physiological traits

Many physiological traits associated with photosynthetic potential efficiency and canopy vigor were measured using portable instruments. Leaf chlorophyll content (SPAD) was measured using a SPAD-502 Plus chlorophyll meter' (Konica Minolta, Japan). Measurements were recorded at the flag leaf during the booting stage (FLSPADB) and grain filling stage (FLSPADGF) between 09:00 and 11:30 under clear sky conditions to minimize diurnal variation. For each plant, three readings (top, middle, and base of the flag leaf) were averaged, and the mean value of five plants was used for plot-level analysis. Stomatal conductance (GS) was measured using an AP4 leaf porometer (Delta-T Devices, Cambridge, UK). Measurements were taken from the abaxial surface of fully expanded flag leaves during the booting stage (FLGSB) and grain filling stage (FLGSGF) between 08:30 and 11:00 under stable environmental conditions. Values were expressed as mmol m^-^² s^-^¹. Normalized Difference Vegetation Index (NDVI) was used to assess canopy greenness and vigor were assessed using a GreenSeeker handheld optical sensor (Trimble Inc., USA). NDVI measurements were taken at roughly 0.8-1.0 m above the canopy, with a consistent orientation and height.

### 2.6 Phenological and yield traits

Phenological and yield components were recorded following standard IRRI evaluation protocols. Days to 50% flowering (DFW) was defined as the number of days from sowing until 50% of the plants within a plot had flowered. The number of productive tillers (NT; count) and grain yield per plant (g plant⁻¹) were determined from five tagged plants at harvest. Grains were oven-dried at 70 °C for 48 h and adjusted to 14% moisture content before determining grain yield. Grain yield per plot (YLD; kg ha⁻¹) was calculated from a harvested area of 5.6 m². Aboveground plant biomass (leaves, panicles, and stems; g plant⁻¹) was determined by oven-drying the harvested plant material at 70 °C until a constant weight was achieved. All physiological devices (SPAD meter, GreenSeeker NDVI sensor, AP4 porometer) were calibrated prior to measurements according to manufacturers guidelines to ensure data accuracy and reproducibility.

#### 2.6.1 Data preprocessing and descriptive analysis

Descriptive statistics were computed using plot-level data for each trait across environments, including mean, standard deviation, coefficient of variation, range and summary are provided in **Table 1**. Trait distributions and seasonal variability were visualized using violin and boxplots. Missing values arising from unbalanced testing across environments were not imputed; instead, analyses were conducted using available observations, and trait availability across season is summarized in **Supplementary Table 2**.

**Table 1:** Summary statistics of representative agronomic and physiological traits across environments.

Trait category	Trait	Mean (range)	SD (range)	CV (%) (range)	Min-max
Phenology	DFW	78.45-86.91	3.61-8.88	4.60-10.22	57-126
Yield traits	Yield	3811-7657	835-2419	12.60-42.19	2257-11923
	YP	26.16-41.71	7.15-11.58	24.65-32.49	13-69.8
Biomass traits	SDW	10.46-24.28	2.72-5.05	13.45-25.98	6.07-36.27
	LDW	7.54-12.91	1.49-3.25	19.52-32.22	2.70-21.33
	PDW	13.07-38.39	3.09-7.51	14.45-27.42	7.37-52.87
Early traits (30 DAS)	PH30	18.17-41.31	1.40-5.25	5.98-12.70	14.97-55.50
	NT30	4.75-10.81	0.48-1.92	9.33-18.05	3.33-15.67
	NDVI30	0.25-0.32	0.05-0.08	20.50-25.58	0.15-0.49
Mid-stage traits (60 DAS)	PH60	62.96-78.59	5.14-7.92	8.01-12.57	52-94
	SPAD60	39.43-41.58	1.74-2.66	4.40-6.74	34.27-46.03
	FLGS60	399.59-465.62	25.88-95.52	5.56-23.49	278-598.67
Physiological traits	NDVI_B	0.69-0.76	0.05-0.06	6.76-8.37	0.59-0.81
	NDVIGF	0.62-0.70	0.03-0.07	4.28-11.68	0.36-0.78

Summary statistics of representative agronomic and physiological traits across environments. Values are presented as ranges across four environments for mean, standard deviation (SD), and coefficient of variation (CV). Minimum and maximum values represent overall observed ranges. Trait abbreviations: DFW, days to 50% flowering; YP, yield per plant; SDW, shoot dry weight; LDW, leaf dry weight; PDW, panicle dry weight; PH30 and PH60, plant height at 30 and 60 DAS; NT30, tiller number at 30 DAS; NDVI, normalized difference vegetation index; SPAD60, chlorophyll content at 60 DAS; FLGS60, stomatal conductance at 60 DA.

#### 2.6.2 Mixed-model analysis and BLUP estimation

To account for the unbalanced multi-environment trial structure and to obtain unbiased genotype estimates, a linear mixed-effects model was fitted for each trait:


$$Y_{ijk}=\mu +E_{j}+G_{i}+(GE{)}_{ij}+R_{k(j)}+{\&\epsilon }_{ijk}$$

where:

$Y_{ijk}$is the observed trait value of genotype i in environment j and replication *k*

μ is the overall mean.

$E_{j}$is the fixed effect of environment.

$G_{i}$is the random effect of genotype.

$(GE{)}_{ij}$ is the genotype × environment interaction.

$R_{k(j)}$is replication nested within environment.

${\&\epsilon }_{ijk}$is the residual error.

Best Linear Unbiased Predictors (BLUPs) were extracted for each genotype and trait. BLUPs were used to remove environmental noise and to obtain genotype-level trait estimates adjusted for environmental and design effects. Because genotype representation differed across environments, BLUP estimation used all available observations without requiring imputation of missing values and accounted for environmental, replication, and genotype × environment effects.

#### 2.6.3 Estimation of repeatability (H²)

Trait repeatability across environments was estimated using variance components derived from the mixed model:


$$H^{2}=\frac{\sigma_{G}^{2}}{\sigma_{G}^{2}+\frac{\sigma_{GE}^{2}}{n_{E}}+\frac{\sigma_{e}^{2}}{n_{E}n_{R}}}$$

where:

$\sigma_{G}^{2}$is genetic variance.

$\sigma_{GE}^{2}$ is genotype × environment variance.

$\sigma_{e}^{2}$is residual variance.

$n_{E}$is the number of environments.

$n_{R}$is the number of replications.

Repeatability was used to assess the reliability and genetic contribution of each trait across environments.

#### 2.6.4 BLUP-based multivariate analysis

Principal component analysis (PCA) and Pearson correlation analysis were conducted using BLUP-adjusted trait values to investigate trait architecture and relationships independent of environmental variation. PCA was used to identify major axes of variation and to assess clustering of genotypes (parents, hybrids, and checks), while correlation analysis was used to quantify relationships between physiological, agronomic, and yield related traits. Details of the PCA loadings are provided in **Supplementary Table 4**.

#### 2.6.5 Season-wise correlation and regression analysis

**Figure 2:**
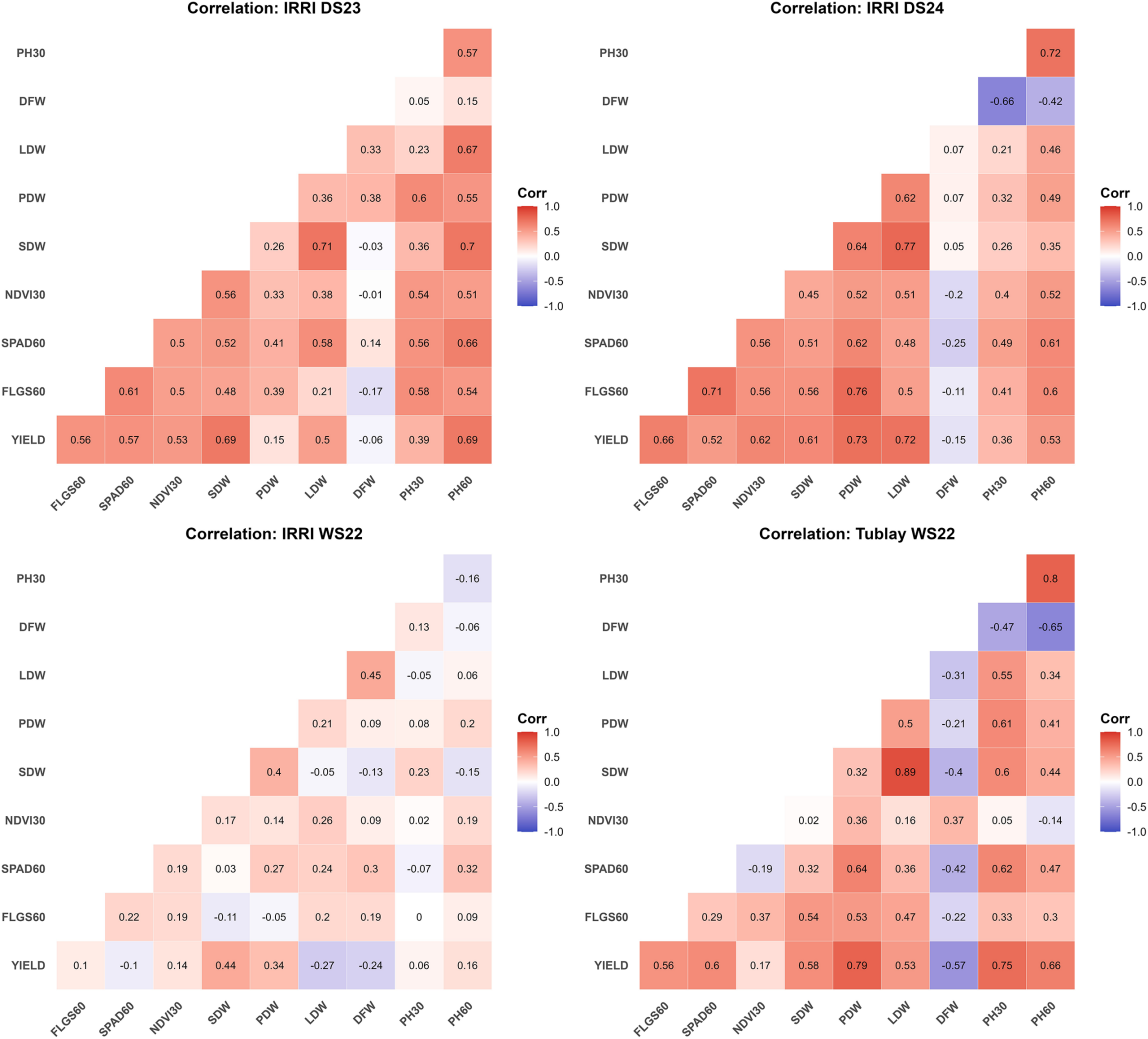
Season-wise correlation between grain yield and key physiological and agronomic traits across four environments. Correlations were computed using genotype mean values within each environment. Stronger and more consistent associations were observed for mid-vegetative traits (60 DAS) compared to early-stage traits.

**Figure 3:**
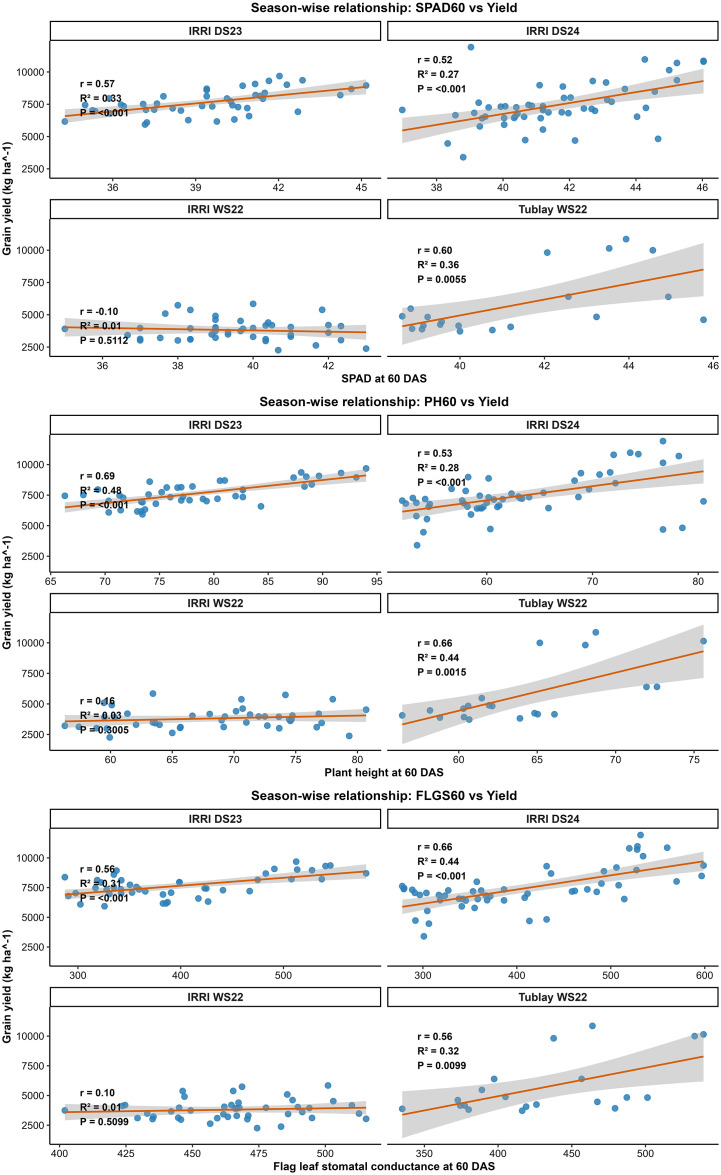
Season-wise relationships between grain yield and key mid-vegetative traits (SPAD60, PH60, and FLGS60). Each panel represents a separate environment, with regression lines indicating the strength and direction of trait-yield relationships. Correlation coefficients (r), coefficients of determination (R²), and significance levels are shown within each panel.

**Figure 4:**
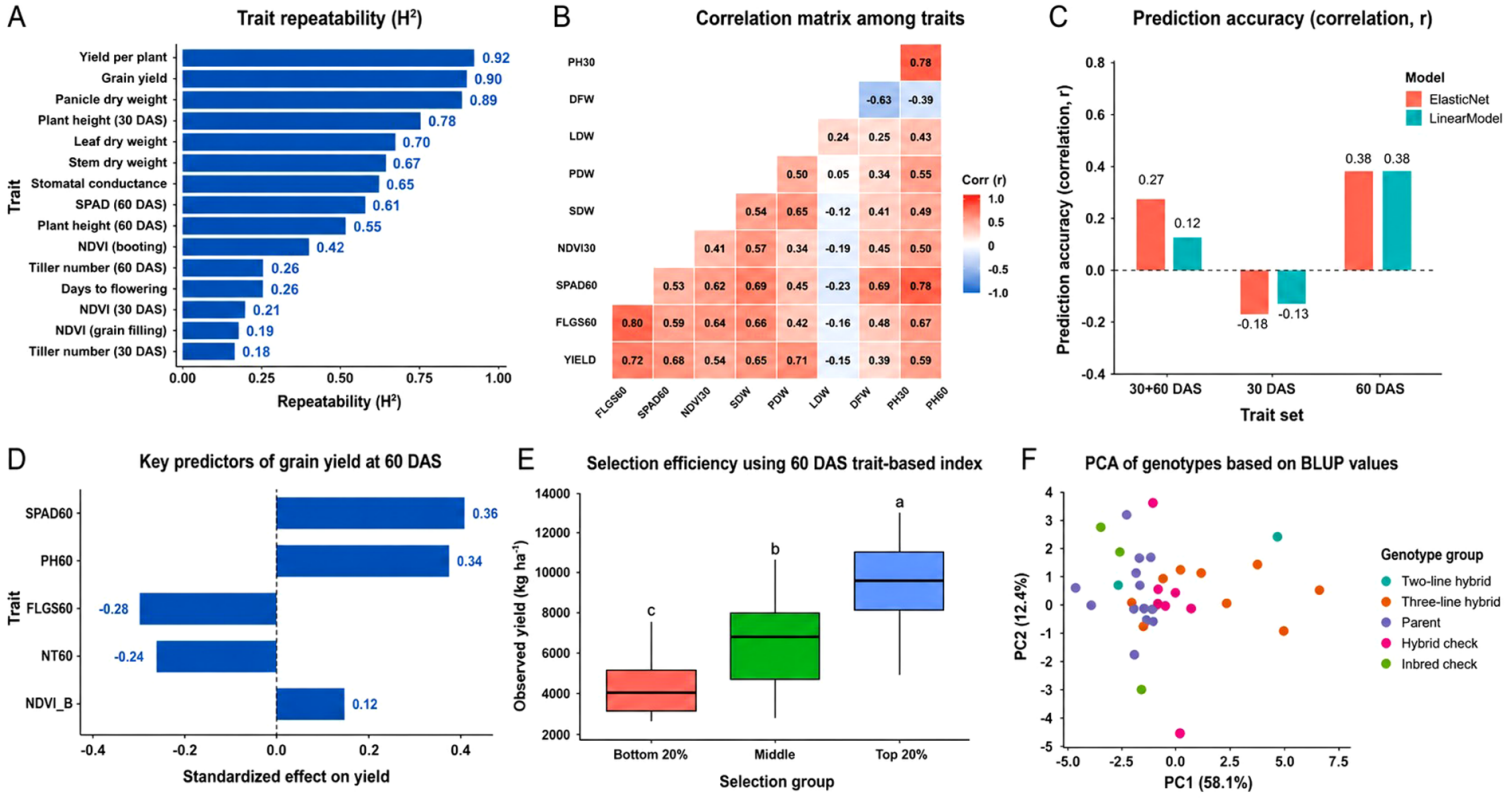
Integrated analysis of trait importance, relationships, and predictive performance for grain yield in hybrid rice across environments. **(A)** Repeatability (broad-sense heritability, H²) of physiological, agronomic, and yield-related traits estimated across multi-environment trials using mixed models. **(B)** Correlation matrix based on BLUP values showing relationships among key traits, including grain yield and selected physiological and agronomic variables. **(C)** Cross-environment prediction accuracy (Pearson’s r) of grain yield using different trait sets (30 DAS, 60 DAS, and combined 30 + 60 DAS), evaluated using linear and elastic net models. **(D)** Standardized regression coefficients indicating the relative importance of selected 60 DAS traits as predictors of grain yield. **(E)** Selection efficiency based on a 60 DAS trait-derived index, showing observed grain yield across genotype groups (bottom 20%, middle, and top 20%). **(F)** Principal component analysis (PCA) of genotypes based on BLUP values, illustrating clustering patterns among genotype groups. All analyses were based on BLUP values obtained from multi-environment mixed models unless otherwise stated. Correlations represent Pearson’s coefficients. Prediction accuracy was assessed using leave-one-season-out cross-validation. Trait abbreviations: SPAD, chlorophyll content; FLGS, flag leaf stomatal conductance; PH, plant height; NT, number of tillers; NDVI, normalized difference vegetation index; DAS, days after sowing.

To evaluate the consistency of trait-yield relationships across environments, season-wise correlation and regression analyses were performed using genotype mean values within each environment (**Figures 2**, **3**). Unlike BLUP-based analyses, which integrate information across environments (**Figure 4B**), these analyses were conducted separately for each season to capture environment-specific relationships. Pearson correlation coefficients were computed between grain yield and key physiological and agronomic traits for each environment independently. This approach allowed assessment of the stability and direction of trait associations under varying environmental conditions. In addition, simple linear regression models were fitted to relate grain yield to selected key predictors (SPAD60, PH60, and FLGS60) for each season. The strength of relationships was evaluated using the Pearson correlation coefficient (*r*), the coefficient of determination (*R²*), and statistical significance (*P*-values). All analyses were conducted using genotype means to avoid the confounding effects of replication and to reflect practical breeding-level selection scenarios.

#### 2.6.6 Cross-environment prediction analysis

To evaluate the predictive ability of early-stage traits, cross-environment prediction was performed using a leave-one-season-out (LOSO) validation strategy. Genotype × season mean values were used to construct the prediction datasets. Three predictor sets were evaluated: (i) traits measured at 30 DAS, (ii) traits measured at 60 DAS, and (iii) a combined set of traits measured at 30 and 60 DAS. Two modeling approaches were implemented, namely linear regression (LM) and Elastic Net regression. In each iteration, one environment was excluded from the test set, and models were trained using data from the remaining environments. Prediction performance was assessed using root mean square error (RMSE), mean absolute error (MAE), and Pearson correlation coefficient (*r*) between observed and predicted grain yield (**Table 2**). This approach enables evaluation of the robustness and generalizability of prediction across environments. Detailed prediction results are provided in **Supplementary Table 5**.

**Table 2:** Prediction performance of grain yield based on physiological traits measured at different growth stages.

Trait set	Model	RMSE	MAE	r
30 DAS	Linear model	7191	6778	-0.13
30 DAS	Elastic net	7074	6668	-0.18
60 DAS	Linear model	3289	3122	0.39
60 DAS	Elastic net	3072	2905	0.38
30 + 60 DAS	Linear model	6534	6096	0.12
30 + 60 DAS	Elastic net	4622	4282	0.27

RMSE, root mean square error; MAE, mean absolute error; r, correlation between observed and predicted yield. Values represent mean performance across environments.

#### 2.6.7 Standardized regression and trait importance

To quantify the relative contribution of individual traits, all predictor variables were standardized (mean = 0, standard deviation = 1), and a multiple linear regression model was fitted using 60 DAS traits. Standardized regression coefficients were used as measures of trait importance, allowing direct comparison of effect sizes across traits.

#### 2.6.8 Development of selection index

The yield prediction index (YPI) was developed using standardized regression coefficients from the 60 DAS model. The index was calculated as a weighted linear combination of standardized trait values. Genotypes were ranked by YPI values and grouped into the top 20%, middle, and bottom 20% categories (**Table 4**; **Figure 4E**). Selection efficiency was evaluated by comparing mean grain yield among these groups and calculating percentage gain relative to the population mean. Details of genotypes ranking based on the selection index are provided in **Supplementary Table 6**.

## 3 Results

### 3.1 Descriptive variation and trait distribution across seasons

**Figure 5:**
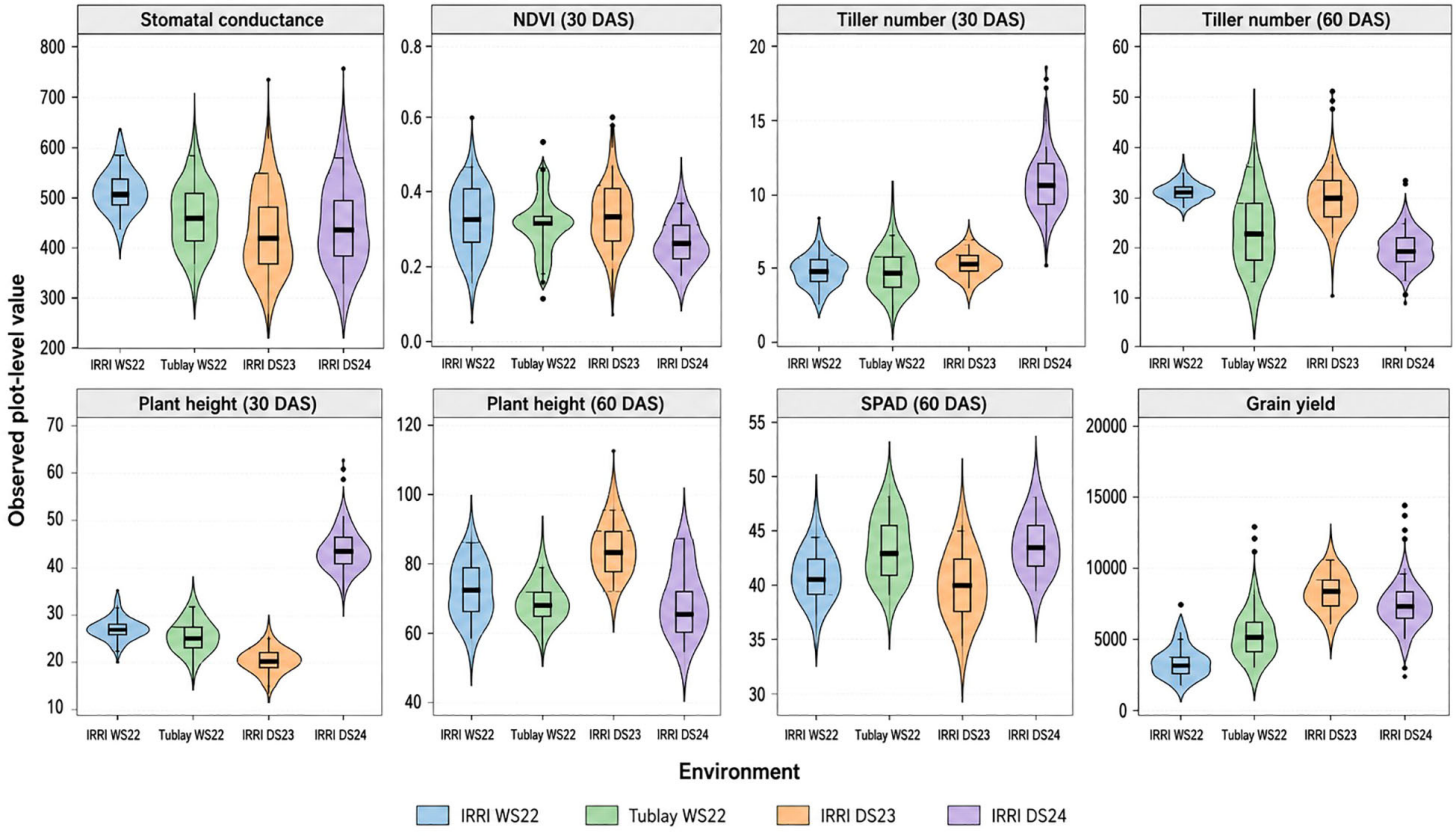
Distribution of key physiological and agronomic traits across contrasting rice-growing environments. Violin plots with embedded boxplots illustrate the distribution of plot-level observations for selected physiological and agronomic traits measured at 30 and 60 days after sowing (DAS) across four cropping environments (IRRI WS2022, Tublay WS2022, IRRI DS2023, and IRRI DS2024). The width of each violin represents the density of observations, whereas the boxplots indicate the median and interquartile range. The figure demonstrates substantial environmental influences on trait expression and reveals that several physiological traits measured at 60 DAS generally exhibited narrower distributions than those measured at 30 DAS, consistent with greater phenotypic stability during the mid-vegetative stage.

Significant variation in trait expression was observed across the four environments, reflecting contrasting seasonal conditions and demonstrating the robustness and suitability of multi-environment analysis (**Figure 5**; **Supplementary Table 2**). Grain yield and biomass-related traits were generally higher in dry-season environments (IRRIDS2023 and IRRIDS2024), whereas wet-season environments showed high variability, particularly for early-stage traits. Early vegetative traits measured at 30 DAS, including PH30, NT30, and NDVI, exhibited broader distributions and higher variability across environments. In contrast, traits measured at 60 DAS, such as PH60, SPAD60, and stomatal conductance (FLGS60), showed comparatively tighter distributions, indicating increased stability at the mid-vegetative stage. These patterns suggest that early-stage measurements are more sensitive to environmental fluctuations, whereas mid-stage traits better capture stabilized plant growth and physiological performance.

### 3.2 Descriptive statistics of trait variability and stability

The descriptive statistic revealed clear differences in variability and stability among trait categories (**Table 1**). Phenological traits, represented by days to 50% DFW, exhibited relatively low variability, with mean values ranging from 78.45 to 86.91 days and CV values between 4.60% and 10.22%, indicating stable expression across the environment. In contrast, yield-related traits showed substantial variation. Grain yield ranged from 3811 to 7657 kg ha^-^¹, with high variability (CV = 12.60-42.19%), reflecting strong environmental influences. Similarly, YP exhibited considerable variation (CV = 24.65-32.49%), indicating the complex and environmentally dependent nature of yield formation. Biomass-related traits, including SDW, LDW, and PDW, showed moderate variability (CV = 13.45–32.22%), suggesting their contribution to yield variation while still being influenced by environmental conditions. Early-stages traits measured at 30 DAS displayed relatively higher variability, particularly NDVI30 (CV = 20.50-25.58%), indicating lower stability and more sensitivity to environmental fluctuation during early growth stage. In contrast, mid-vegetative traits measured at 60 DAS exhibited reduced values; notably, SPAD60 showed a very low CV (4.40-6.74%), while PH60 ranged between 8.01% and 12.57%, indicating stability and consistency across the environment. Although FLGS60 indicated a wider ranged (CV = 5.56-23.49%), it reflected improved stability compared to early stages-traits. Physiological traits further supported this pattern, with NDVI_B (CV = 6.76-8.37%) and NDVIGF (CV = 4.28-11.68%) showing moderate to high stability across environments.

### 3.3 Variance components and heritability

Variance component analysis revealed substantial differences among traits in the relative contributions of genetic, genotype × environment (G×E), and residual effects (**Supplementary Table 2**). Grain yield exhibited a large genetic variance (σ²g = 1,595,135.2) compared to G×E variance (σ²g×e = 363,577.4) and residual variance (σ²e = 216,733.1), resulting in high broad-sense heritability (H² = 0.93). Similarly, YP showed very high heritability (H² = 0.95), indicating strong genetic control. Biomass-related traits such as panicle dry weight (PDW; H² = 0.92), shoot dry weight (SDW; H² = 0.76), and leaf dry weight (LDW; H² = 0.77) also exhibited moderate to high heritability, suggesting that these traits are largely governed by genetic factors with relatively lower environmental influence. In contrast, phenological traits such as DFW showed low heritability (H² = 0.29), primarily due to a large G×E component (σ²g×e = 13.83) relative to genetic variance (σ²g = 2.21), indicating strong environmental sensitivity. Early-stage traits exhibited lower heritability, particularly NDVI30 (H² = 0.25) and NT30 (H² = 0.00), reflecting high environmental noise and limited genetic control at early growth stages. In contrast, mid-vegetative traits such as SPAD60 (H² = 0.71) and FLGS60 (H² = 0.74) showed moderate to high heritability, showing genetic stability control. Structural traits showed intermediate patterns, with PH30 exhibiting relatively high heritability (H² = 0.82), while PH60 showed moderate heritability (H² = 0.66), suggesting increasing environmental influence at later stages. Some variance components, particularly for early-stage traits such as NT30, were estimated as zero, indicating negligible genetic variation and strong environmental influence at early developmental stages (**Supplementary Table 2**). The high heritability observed for yield per plant indicates that genetic differences among genotypes explained a large proportion of the phenotypic variation under the evaluated environments. This does not imply universal heritability across all environments but reflects the variance structure of the present multi-environment experiment. Traits such as YP, PDW, and Yield exhibited relatively high heritability, suggesting strong genetic determination under the evaluated environments, whereas traits including NT30, NT60, and NDVI30 showed lower heritability, reflecting environmental influence and developmental plasticity.

### 3.4 Genetic basis and repeatability of physiological and agronomic traits

Variance component analysis revealed substantial genetic variation for key traits across environments (**Table 2**; **Figure 4A**). The YLD, YP, and PDW exhibited high repeatability, indicating strong and consistent genetic control.

Among early-stage traits, moderate repeatability was observed for PH30 and NDVI-related measurements, whereas NT30 showed relatively lower repeatability, reflecting higher environmental sensitivity. At 60 DAS, several traits displayed moderate-to-high repeatability, including SPAD60, PH60, and FLGS60, indicating that these traits are not only biologically relevant but also genetically stable across environments. Overall, these results demonstrate that mid-vegetative physiological and structural traits provide a more reliable genetic signal compared to early-stage measurements.

### 3.5 BLUP-based trait architecture and genotype differentiation

Principal component analysis (PCA) based on BLUP-adjusted values revealed clear structuring of trait variation **Figure 4F**; **Supplementary Table 4**. The first principal component (PC1) explaining ~58% of total variation, was strongly associated with key yield related trait, including SPAD60, PH60, and BM components, indicating that this axis represent overall physiological efficiency and growth potential. Hybrid genotypes were predominantly distributed alongside of PC1, reflecting increased physiological and structural vigor, whereas parental lines clustered closer to the origin or negative side, indicating comparatively lower productivity, this separation highlights the multivariate nature of heterosis, where hybrid integrate multiple favorable physiological traits. The second principal component (PC2), explaining ~12% of variation, captured secondary traits variation, potentially related to phenology and canopy structure. Checks occupied an intermediate position, suggesting moderate performance relative to hybrids (**Figure 4F**). Trait loadings for the principal components are presented in **Supplementary Table 4**.

### 3.6 Relationships between physiological traits and grain yield

BLUP-based correlation analysis revealed a positive and biologically meaningful association between yield and several physiological and agronomic traits (**Figure 4B**). Grain yield showed strong positive correlations with YP, PDW, PH60, and SPAD60. Indicating that both photosynthetic potential efficiency and BM accumulation are key determinant of productivity. Notably, SPAD60 exhibited one of the strongest correlations with yield, highlighting the importance of chlorophyll-mediated photosynthetic potential efficiency. Stomatal conductance (FLGS60) also showed a positive association with yield, supporting its role in regulating carbon assimilation through gas exchange. In contrast, DFW exhibited negative correlations with yield, indicating that earlier flowering genotypes achieve higher productivity under the tested conditions. NDVI and NT showed moderate correlations with yield, suggesting that while canopy vigor contributes to productivity, it is not sufficient alone to fully explain yield variation. The correlation structure demonstrates that yield is governed by an integrated network of physiological efficiency and structural development rather than single trait effects. A complete BLUP-based correlation matrix is provided in **Supplementary Table 3**.

### 3.7 Environment- specific stability of trait-yield relationship

To further evaluate the consistency of trait-yield relationships across contrasting environments, season-wise correlation and regression analyses were conducted using genotype means within each season (**Figures 2**, **3**). These analyses provided environment-specific insights that complement the across-environment BLUP-based results. Across environments, traits measured at 60 DAS consistently showed stronger and more stable associations with grain yield compared to early-stage (30 DAS) traits, indicating the increasing importance of plant functioning mid-vegetative development. In the IRRI dry seasons (DS2023 and DS2024), grain yield exhibited moderate to strong positive correlations with SPAD60 (r ≈ 0.52-0.57), PH60 (r ≈ 0.53-0.69), and FLGS60 (r ≈ 0.56-0.66). These results suggest that both physiological efficiency (chlorophyll content and stomatal conductance) and structural growth (plant height) contribute substantially to yield formation under relatively favorable and stable environmental conditions. In contrast, wet-season environments displayed more variable trait-yield relationships. In IRRI WS2022, correlations between grain yield and most physiological traits were weak or non-significant, reflecting environmental variability and reduced signal strength under more fluctuating field conditions. However, in the cooler Tublay WS2022 environment, moderate to strong positive associations were observed, particularly for SPAD60 (r ≈ 0.60) and PH60 (r ≈ 0.66), indicating that more stable or less stressful climatic conditions can enhance the expression and detectability of biologically relevant trait-yield relationships even within wet seasons. Season-wise regression analyses further supported these patterns. In dry seasons, SPAD60, PH60, and FLGS60 consistently exhibited positive linear relationships with grain yield, explaining a substantial proportion of yield variation (R² ≈ 0.27-0.48). In contrast, regression strength was markedly reduced in IRRI WS2022, where relationships were weak and, in some cases, non-significant, consistent with the correlation analysis. Notably, regression performance improved in the Tublay WS2022 environment (R² ≈ 0.32-0.44), reinforcing the influence of environmental context on trait-yield linkage strength.

Collectively, these findings indicate that mid-vegetative traits, particularly SPAD60 and PH60, serve as robust and biologically meaningful predictors of grain yield across environments. However, the magnitude of their effects is environment-dependent, highlighting the importance of considering seasonal context when interpreting trait-yield relationships in hybrid rice.

### 3.8 Identification of the optimal phenotyping stage for yield prediction

Cross-environment prediction analysis revealed clear differences in predictive performance among trait sets (**Figure 4C**; **Table 2**). Traits measured at 30 DAS showed poor predictive ability, with low or negative correlations between observed and predicted yield across environments. In contrast, 60 DAS traits provided the highest prediction accuracy, with correlation coefficients reaching approximately 0.38 across validation environments for both linear and Elastic Net models. Notably, combining 30 DAS and 60 DAS traits did not improve prediction performance and, in some cases, reduced accuracy. These findings demonstrate that early-stage traits alone are insufficient for reliable yield prediction, whereas mid-vegetative stage traits capture the critical physiological processes underlying yield formation. Thus, 60 DAS represents the optimal phenotyping stage for predicting grain yield across environments.

### 3.9 Relative contribution of physiological and agronomic predictors at 60 DAS

Standardized regression analysis of 60 DAS traits revealed distinct contributions of individual predictors to grain yield (**Table 3**; **Figure 4D**). SPAD60 and PH60 exhibited the strongest positive effects, indicating that photosynthetic potential and structural vigor are key regulators of yield. FLGS60 and NT60 showed weak or context-dependent effects, suggesting potential trade-offs in resource allocation under certain environmental conditions. NDVI at booting stage (NDVI_B) showed a relatively weak contribution. Comprehensive prediction metrics for each environment and model are provided in **Supplementary Table S5**.

**Table 3:** Standardized regression coefficients for physiological traits measured at 60 DAS and their relative contributions to grain yield.

Trait	Estimate	SE	t value	p value
SPAD60	0.46	0.12	3.7	<0.001
PH60	0.45	0.11	3.97	<0.001
FLGS60	-0.27	0.14	-1.97	0.053
NT60	-0.24	0.13	-1.93	0.058
NDVI_B	0.13	0.12	1.12	0.27

Estimates represent standardized regression coefficients obtained from a multiple linear regression model using physiological traits measured at 60 DAS. SE, standard error of the regression coefficient; t, t-statistic; P, probability value.

#### 3.9.1 Practical application: selection efficiency of the 60 DAS index

To evaluate the practical utility of the identified predictors, a selection index was developed using standardized coefficients from the 60 DAS model. Genotypes were ranked based on index values and grouped into top 20%, middle, and bottom 20% classes. The top 20% of genotypes exhibited a mean grain yield of 8491.92 kg ha^-^¹, compared with 6105.57 kg ha^-^¹ for the population mean and 4343.49 kg ha^-^¹ for the bottom 20% (**Table 4**; **Figure 4E**). This corresponds to a selection gain of 39.08% over the population mean. The clear separation among selection groups and the substantial yield advantage of the top group demonstrate that the 60 DAS trait-based index provides an effective and practical tool for early-stage selection in hybrid rice breeding.

**Table 4:** Summary of the selection index showing the mean grain yield of the top 20% selected genotypes, the bottom 20% selected genotypes, the overall population mean, and the estimated selection gain.

Selection group	Yield	Unit
Top 20%	8491.92	kg ha-1
Bottom 20%	4343.49	kg ha-1
Population mean	6105.57	kg ha-1
Selection gain	39.08	%

Grain yield is expressed as kg ha^-1^ Selection gain (%) was calculated as the percentage increase in the mean grain yield of the top 20% selected genotypes relative to the overall population mean.

## 4 Discussion

### 4.1 Mid-vegetative stage as the optimal stage for yield prediction in rice hybrid

**Figure 6:**
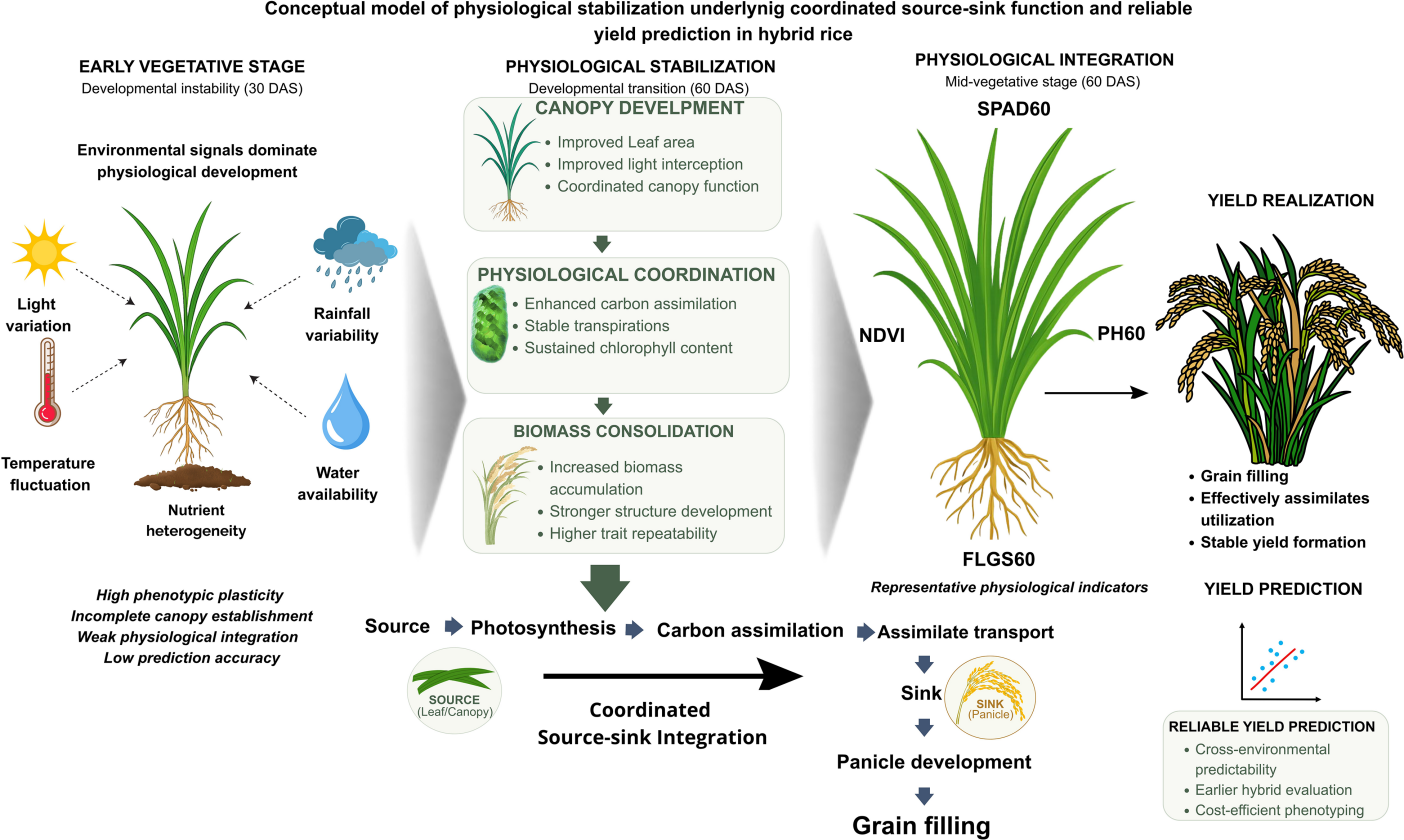
Conceptual framework illustrating physiological stabilization as the basis for coordinated source-sink function and reliable yield prediction in hybrid rice. The model summarizes the developmental transition from the environmentally responsive early vegetative stage (30 DAS) to the physiologically stabilized mid-vegetative stage (60 DAS), where improved canopy development, photosynthetic coordination, and carbon assimilation enhance source-sink integration. This coordinated physiological state promotes biomass accumulation, grain filling, and stable yield formation, while key physiological traits (SPAD60, PH60, FLGS60, and NDVI) provide reliable indicators for cross-environment yield prediction and early hybrid selection.

A central finding of this study is that traits measured at 60 DAS relatively outperformed those measured at 30 DAS for predicting grain yield across environments. Early-stage traits (30 DAS) showed weak and inconsistent predictive ability, whereas mid-vegetative traits (60 DAS) provided moderate but stable prediction accuracy across environments. To summarize the biological interpretation emerging from the present study, a conceptual framework is proposed (**Figure 6**). The framework illustrates the developmental transition from early vegetative plasticity (30 DAS) to physiological stabilization at the mid-vegetative stage (60 DAS), highlighting how canopy development, physiological coordination, and source-sink integration collectively contribute to reliable cross-environment prediction of grain yield in hybrid rice. At early vegetative stages, plant growth is highly sensitive to transient environmental conditions, and phenotypic expression is dominated by short-term fluctuations rather than stable physiological processes (Schurr et al., 2006; Bahuguna and Jagadish, 2015). In contrast, by the maximum tillering stage, canopy structure, source-sink dynamics, and biomass accumulation begin to stabilize, making trait expression more representative of the plant’s intrinsic productivity potential (Larue et al., 2019; Luxmoore, 1991). Previous studies have emphasized that the timing of phenotyping critically influences the predictive value of traits, particularly under field conditions where environmental variability is high (Araus and Cairns, 2014; Furbank and Tester, 2011). The present results extend this understanding by demonstrating, in a multi-environment rice hybrid system, that the mid-vegetative stage represents a biologically meaningful and practically optimal phenotyping phase (Cowley et al., 2014; Reynolds et al., 2012; Tucker et al., 2020). The environmental patterns observed across seasons (**Figure 6**) provide important context for the variability in trait-yield relationships. The high rainfall variability and extreme events during IRRI WS2022 likely introduced environmental noise, reducing the stability of physiological trait expression and weakening their association with grain yield. In contrast, the more stable conditions observed during the dry seasons allowed clearer expression of physiological and structural traits, resulting in stronger and more consistent relationships with yield. The relatively cooler and stable conditions in Tublay further supported this pattern. These findings suggest that environmental stability plays a critical role in modulating heterosis expression, with favorable, stable conditions enabling the physiological advantages of hybrid genotypes to be realized more consistently. Thus, the lower predictive value of 30 DAS traits should not be interpreted simply as poor trait performance, but as evidence that early vegetative physiology had not yet become developmentally integrated. At this stage, canopy establishment, biomass accumulation, and source-sink coordination were still incomplete, making trait expression more vulnerable to environmental fluctuation. By contrast, 60 DAS appears to represent a physiological stabilization phase in which canopy function, structural growth, and assimilate production become sufficiently coordinated to provide more reliable signals of final yield potential.

### 4.2 Physiological determinants of yield: the central role of photosynthetic efficiency

Among the evaluated phenotypic and agronomic traits, SPAD60 and PH60 emerged as the strongest positive predictors of yield, while stomatal conductance FLGS60 also contributed significantly. These results highlight the importance of physiological efficiency and structural development in determining grain yield. Chlorophyll content is closely associated with leaf nitrogen status and photosynthetic potential, which are key drivers of biomass accumulation and grain production (Evans, 2013; Long et al., 2006; Qu et al., 2017). Although SPAD does not directly measure photosynthetic rate or carbon assimilation, it serves as a practical field indicator of chlorophyll status and leaf nitrogen-related greenness. Consequently, the strong association of SPAD60 with grain yield suggests that hybrids maintaining higher chlorophyll levels during the mid-vegetative stage are better able to sustain canopy photosynthesis, promoting biomass accumulation and ultimately enhancing grain yield. Likewise, FLGS60 represents leaf-level gas exchange, linking stomatal regulation with carbon assimilation and water use under field conditions (Lawson and Blatt, 2014; Wang et al., 2022; Nguyen et al., 2023). Importantly, the convergence of evidence across BLUP-based, season-wise, and regression-based analyses strengthens the interpretation that the identified 60 DAS traits are not merely statistical correlates but biologically meaningful expressions of hybrid vigor. The BLUP-based PCA and correlation structure showed that hybrids occupied a distinct multivariate space associated with higher productivity, indicating that heterosis was expressed through coordinated gains in physiological efficiency and structural growth. This interpretation was reinforced by season-wise genotype-mean analyses, in which SPAD60, PH60, and FLGS60 repeatedly maintained positive relationships with grain yield, especially under the more stable dry-season environments. Standardized regression further narrowed this signal to a small set of mid-vegetative traits with practical predictive value, with SPAD60 and PH60 emerging as the most consistent contributors. Together, these results suggest that heterosis in this hybrid rice panel is expressed most clearly at the mid-vegetative stage, when canopy function, source strength, and structural vigor begin to converge into a stable yield-forming phenotype (Hatfield and Prueger, 2010; Tucker et al., 2020; Hall and Richards, 2013).

### 4.3 Structural traits and their interaction with physiological processes

While physiological traits showed the strongest direct effects, structural traits such as tiller number (NT60) and NDVI exhibited weaker or context-dependent contributions to yield. In the present study, tiller number showed a marginal negative association with yield in the standardized model, suggesting potential trade-offs between tiller formation and resource allocation. This observation is consistent with previous findings indicating that excessive tillering can lead to competition for assimilates, resulting in reduced grain filling efficiency (Peng et al., 2004; Sheehy et al., 2006). Similarly, NDVI, which captures general canopy greenness and biomass, showed only moderate predictive value, indicating that canopy vigor alone is insufficient to fully explain yield variation (Wahab et al., 2018; Hassan et al., 2019; Miller et al., 2018). These results highlight that yield formation is not determined by individual traits in isolation but rather by the integration of physiological efficiency and optimized structural development (Mathan et al., 2016; Sinclair and Vadez, 2002). Traits that contribute to yield must therefore be interpreted within the broader context of resource allocation and plant architecture. These findings support the interpretation that structural traits contribute to yield only when they are physiologically coordinated with source activity and sink demand. In this context, higher vegetative growth is beneficial only if it supports carbon acquisition, biomass consolidation, and efficient allocation of assimilates toward developing reproductive sinks.

### 4.4 Physiological basis of heterosis: insights from multivariate trait structure

The BLUP-based PCA revealed clear separation between hybrids and parental lines along the primary axis of variation, which was strongly associated with productivity-related traits. This supports the concept that heterosis in hybrid rice is expressed as a coordinated enhancement of multiple physiological and structural traits rather than a single dominant factor. Hybrids exhibited higher values for traits associated with photosynthetic potential, biomass accumulation, and canopy development, consistent with previous reports that hybrid vigor is linked to improved carbon assimilation and resource-use efficiency (Chen, 2013; Huang et al., 2016b; Meena et al., 2021; Huang et al., 2016a). The intermediate positioning of checks further indicates that elite hybrids occupy a distinct physiological space characterized by enhanced performance. This separation suggests that hybrid superiority was expressed as a coordinated physiological state rather than as the enhancement of one isolated trait. In other words, heterosis in the evaluated panel appears to emerge through the combined expression of canopy development, source strength, biomass accumulation, and sink-supporting architecture. These findings reinforce the view that heterosis arises from the integration of multiple biological processes, including enhanced photosynthesis, improved sink strength, and optimized source-sink relationships (Blum, 2013; Smith et al., 2018; Chang and Zhu, 2017). Importantly, the present study demonstrates that these processes become most evident at the mid-vegetative stage, providing a practical gap for their evaluation.

### 4.5 Importance of BLUP-based analysis in unbalanced multi-environment trials

The use of BLUPs was critical for extracting reliable genotype-level information from an unbalanced multi-environment dataset (Muir, 2007; Habier et al., 2013; Quinton et al., 1992). In breeding trials, genotypes are typically evaluated across multiple environments and under different levels of replication, making direct comparisons based on raw means unreliable. BLUPs provide adjusted estimates by accounting for environmental effects, replication, and genotype × environment interactions, thereby improving the accuracy and comparability of trait values (Piepho et al., 2008; Crossa et al., 2006; Balzarini et al., 2024). This is particularly important when integrating data across seasons and locations, as in the present study. Furthermore, the use of BLUP-adjusted values in PCA, correlation, and prediction analyses ensures that observed relationships reflect underlying biological patterns rather than confounding environmental effects. This methodological approach strengthens the robustness of the conclusions and enhances their relevance for breeding applications.

### 4.6 Cross-environment prediction and its implications for breeding

The use of leave-one-season-out validation demonstrated that predictive relationships derived from one set of environments can be generalized to others, although with moderate accuracy. This is a more stringent and realistic evaluation compared to within-environment validation and reflects the challenges of predicting performance under variable field conditions. The finding that 60 DAS traits provide the most reliable cross-environment prediction has important implications for breeding programs. It suggests that phenotyping efforts can be strategically focused on this stage to maximize predictive efficiency while minimizing resource use. Moreover, the moderate prediction accuracy observed in this study is consistent with the complex and polygenic nature of yield (Cooper et al., 2014; Ma and Zhou, 2021; Shahi et al., 2022; Guo et al., 2020), which is influenced by numerous interacting factors. Rather than aiming for perfect prediction, the goal is to identify robust and biologically meaningful indicators that can guide selection decisions. Although the observed prediction accuracy was moderate, this is biologically reasonable because grain yield is a highly complex and environmentally sensitive trait. The value of the model, therefore, lies not in perfect prediction but in identifying developmentally stable and operationally feasible physiological indicators that can support earlier selection decisions across environments.

### 4.7 Practical utility of the selection index for early-stage breeding decisions

The 60 DAS trait-based selection index resulted in a substantial yield gain (39.08%), demonstrating its practical values for early-stage selection. The clear separation between top and bottom selection groups indicates that the index captures meaningful variation in productivity and can be used to identify superior genotypes at an earlier stage of development. Similar approaches have been shown to enhance selection efficiency by integrating multiple physiological traits into a unified index (Reynolds and Langridge, 2016; Araus et al., 2018). This has significant implications for breeding efficiency, as it allows for earlier selection and reduces the need for extensive end-season evaluation (Escamilla et al., 2025; Yuan et al., 2019). Importantly, the index integrates multiple traits, reflecting the complex nature of yield determination, while remaining simple enough for practical application. This balance between biological relevance and operational simplicity is essential for successful adoption in breeding programs. From a breeding perspective, this framework may reduce dependence on labor-intensive late-season evaluation by focusing phenotyping on a biologically informative stage. Such physiology-guided screening could be particularly useful in large hybrid evaluation programs where rapid, non-destructive, and cost-efficient indicators are needed before final yield assessment.

### 4.8 Implications for two-line and three-line hybrid systems

Both two-line and three-line hybrids were positioned in the high-performing region of the PCA, indicating that the physiological determinants of yield identified in this study are broadly applicable across hybrid systems. While the genetic mechanisms underlying sterility differ between these systems, the physiological processes governing yield formation appear to converge. This suggests that the proposed phenotyping framework and selection index can be applied across hybrid breeding platforms, providing a practical approach for integrating physiological traits into the hybrid rice improvement program. Together, these findings demonstrate that heterosis in hybrid rice is expressed through coordinated physiological processes that become most evident at the mid-vegetative stage, where plant growth, canopy function, and resource-use efficiency converge into a stable yield-forming phenotype. This convergence suggests that although two-line and three-line hybrids differ in reproductive control systems, their yield formation may depend on shared physiological processes once canopy function and source-sink coordination become stabilized.

### 4.9 Limitation and future perspectives

While the present study identifies 60 DAS as a biologically informative stage for physiology-guided yield prediction, several limitations should be acknowledged. First, SPAD, NDVI, plant height, and stomatal conductance provide practical field-based indicators, but they do not fully capture whole-canopy photosynthesis, flag leaf architecture, or carbohydrate synthesis during grain filling. Future studies, including direct photosynthetic measurements, flag leaf area, leaf angle, and reproductive-stage physiology, would provide deeper mechanistic resolution. Second, early-stage stomatal conductance was not available across all environments; therefore, its potential role in seedling vigor and early growth prediction remains an important area for further evaluation. Third, integrating molecular predictors, such as parental genetic distance or genomic marker information, may improve predictive accuracy when combined with physiological traits. Finally, although mixed-model BLUPs helped accommodate unequal replication and unbalanced genotype representation, broader validation across larger hybrid panels and additional environments will be necessary to confirm the general applicability of this framework.

## 5 Conclusion

This study demonstrates that the mid-vegetative stage represents a critical developmental phase for capturing physiologically integrated signals of heterosis and yield potential in hybrid rice. Traits measured at 60 DAS, particularly SPAD60 and PH60, provided a stronger and more consistent prediction of grain yield across environments than early-stage measurements, indicating that physiological stabilization, canopy development, biomass consolidation, and source-sink coordination become more informative at this stage. Despite differences in hybrid systems, high-performing two-line and three-line hybrids shared similar physiological characteristics during the mid-vegetative stage, suggesting that common physiological mechanisms underpin yield formation across both breeding platforms. By integrating multi-environment analysis, BLUP-based trait estimation, and cross-environment prediction, this work provides a practical framework for physiology-guided phenotyping and early hybrid evaluation. These findings support the use of biologically meaningful developmental stages to improve selection efficiency, reduce late-season phenotyping burden, and strengthen breeding decisions in hybrid rice improvement programs.

## Data Availability

The original contributions presented in the study are included in the article/**Supplementary Material**. Further inquiries can be directed to the corresponding author.

## Supplementary material

Supplementary Table 1 Seasonal climate data and soil properties Trait definitions, abbreviations, units, and measurement stages.

Supplementary Table 2 Season-wise descriptive statistics for all traits.

Supplementary Table 3 Full BLUP-based correlation matrix.

Supplementary Table 4 Principal component loadings.

Supplementary Table 5 Detailed prediction results across environments and models.

Supplementary Table 6 Complete genotype ranking based on selection index.
